# Understanding the effects of universal test and treat on longitudinal HIV care outcomes among South African youth: a retrospective cohort study

**DOI:** 10.1186/s12889-023-16353-9

**Published:** 2023-09-05

**Authors:** Lindsey M. Filiatreau, Jessie K. Edwards, Nkosinathi Masilela, F. Xavier Gómez-Olivé, Nicole Haberland, Brian W. Pence, Joanna Maselko, Kathryn E. Muessig, Chodziwadziwa Whiteson Kabudula, Mi-Suk Kang Dufour, Sheri A. Lippman, Kathleen Kahn, Audrey Pettifor

**Affiliations:** 1 Division of Infectious Diseases, School of Medicine, Washington University in St. Louis, St. Louis, USA; 2 Department of Epidemiology, Gillings School of Global Public Health, University of North Carolina at Chapel Hill, Chapel Hill, NC USA; 3 MRC/Wits Rural Public Health and Health Transitions Research Unit (Agincourt), School of Public Health, Faculty of Health Sciences, University of the Witwatersrand, Johannesburg, South Africa; 4 Population Council, New York, NY USA; 5 College of Nursing, Florida State University, Tallahassee, FL USA; 6 Biostatistics Division, School of Public Health, University of California, Berkeley, Berkeley, CA USA; 7 Division of Prevention Science, Department of Medicine, University of California, San Francisco, CA USA; 8 Carolina Population Center, Chapel Hill, NC USA

**Keywords:** Universal test and treat, HIV care continuum, Youth living with HIV, South Africa, Linkage to care, Viral suppression, Retention in care

## Abstract

**Introduction:**

Little is known about the effects of universal test and treat (UTT) policies on HIV care outcomes among youth living with HIV (YLHIV). Moreover, there is a paucity of information regarding when YLHIV are most susceptible to disengagement from care under the newest treatment guidelines. The longitudinal HIV care continuum is an underutilized tool that can provide a holistic understanding of population-level HIV care trajectories and be used to compare treatment outcomes across groups. We aimed to explore effects of the UTT policy on longitudinal outcomes among South African YLHIV and identify temporally precise opportunities for re-engaging this priority population in the UTT era.

**Methods:**

Using medical record data, we conducted a retrospective cohort study among youth aged 18–24 diagnosed with HIV from August 2015-December 2018 in nine health care facilities in South Africa. We used Fine and Gray sub-distribution proportional hazards models to characterize longitudinal care continuum outcomes in the population overall and stratified by treatment era of diagnosis. We estimated the proportion of individuals in each stage of the continuum over time and the restricted mean time spent in each stage in the first year following diagnosis. Sub-group estimates were compared using differences.

**Results:**

A total of 420 YLHIV were included. By day 365 following diagnosis, just 23% of individuals had no 90-or-more-day lapse in care and were virally suppressed. Those diagnosed in the UTT era spent less time as ART-naïve (mean difference=-19.3 days; 95% CI: -27.7, -10.9) and more time virally suppressed (mean difference = 17.7; 95% CI: 1.0, 34.4) compared to those diagnosed pre-UTT. Most individuals who were diagnosed in the UTT era and experienced a 90-or-more-day lapse in care disengaged between diagnosis and linkage to care or ART initiation and viral suppression.

**Conclusions:**

Implementation of UTT yielded modest improvements in time spent on ART and virally suppressed among South African YLHIV— however, meeting UNAIDS’ 95-95-95 targets remains a challenge. Retention in care and re-engagement interventions that can be implemented between diagnosis and linkage to care and between ART initiation and viral suppression (e.g., longitudinal counseling) may be particularly important to improving care outcomes among South African YLHIV in the UTT era.

**Supplementary Information:**

The online version contains supplementary material available at 10.1186/s12889-023-16353-9.

## Introduction

Traditional methods for exploring HIV treatment and care outcomes (e.g., cross-sectional HIV care continuums) fail to capture the complexity of the processes whereby people enter and leave HIV care, start and stop anti-retroviral therapy (ART), and alternate between being virally-suppressed and non-suppressed over time [1]. As depicted by Ehrenkranz et al., individuals may disengage from care (i.e., stop attending HIV clinic visits), and subsequently reengage, at any point along the traditional HIV care continuum [1]. Often, this process of cycling in and out of care occurs multiple times across an individual’s care trajectory, thereby impeding consistent ART use necessary for achieving sustained viral suppression.

Longitudinal HIV care continuum frameworks, including that formalized by Ehrenkranz and colleagues, have been proposed as an alternative to the traditional cross-sectional cascade as they provide a more nuanced picture of population-level care engagement outcomes over time [2, 3]. These longitudinal methods may be particularly useful for identifying specific gaps in treatment and care services at precise time points following diagnosis, and for elucidating which factors truly impact HIV care outcomes across the cascade [2, 4].

Existing evidence suggests youth living with HIV (YLHIV) experience worse HIV treatment outcomes at each stage in the HIV care continuum compared to adults [4–8]. In sub-Saharan Africa, YLHIV are particularly vulnerable to suboptimal treatment and care outcomes [7, 9]. A 2016 meta-analysis conducted by Zanoni and colleagues found that just 14% of South African YLHIV ages 15 to 24 accessed ART [10]. Among those who accessed ART, an estimated 83% were retained in care and 81% were virally suppressed, yielding an overall prevalence of suppression of 10% [10]. These estimates make clear the reality that access to ART was one of the largest barriers to achieving viral suppression among South African YLHIV prior to 2016.

In line with the World Health Organization’s treatment recommendations, the South African government adopted a universal test and treat (UTT) policy in September 2016 increasing access to ART for all people living with HIV regardless of clinical stage [12]. While some studies suggest this policy change yielded improvements in multiple HIV care outcomes among South African adults living with HIV, others suggest it increased attrition from care following treatment initiation [11, 12]. Importantly, this policy change held the potential to overcome the primary barrier to achievement of viral suppression among YLHIV identified by Zanoni and colleagues [10]. However, little remains known about the true effects of this policy on longitudinal care outcomes among YLHIV specifically [10, 13]. Current cross-sectional data suggest poor retention in care and viral non-suppression persist among YLHIV even in the era of UTT [13, 14].

Addressing barriers to sustained engagement care among YLHIV is critical if we are to end the HIV epidemic by 2030 [9, 18]– yet, a limited number of studies have identified temporally precise opportunities for re-engaging YLHIV following lapses in care in the UTT era [6]. To address the identified gaps in the literature, we draw on the longitudinal HIV care continuum framework to: (1) explore the effects of the UTT policy on HIV care outcomes among YLHIV in rural South Africa, and (2) identify temporally precise opportunities for re-engaging this priority population in HIV care during the UTT treatment era.

## Methods

### Study site

This study was conducted in the Agincourt Health and Socio-Demographic Surveillance System study area (HDSS) in rural Mpumalanga Province, South Africa [15]. This area is approximately 500 km northeast of Johannesburg [15] and home to nearly 120,000 individuals [16]. An estimated 27% of young women and 6% of young men ages 20 to 24 in this area are living with HIV, consistent with national trends that suggest young women are over three times as likely to be living with HIV compared to their male counterparts [17]. Access to public sector services and economic opportunities post-schooling in the HDSS is limited, contributing to a high degree of work-related migration, particularly among youth exiting the school system [18].

Nine publicly funded health care facilities provide medical services to a majority of study area residents [19]. Within these facilities, access to primary health care, HIV counseling and testing services, and ART is free of charge. Across the study period, nationally recommended HIV counseling and testing services consisted of pre-test group and individual information sessions followed by testing and post-test counseling [20]. Patient wait times in this setting often exceed national standards and there is limited to no differentiated care for young people [21].

### Study population

We extracted data for all individuals aged 18 to 24 with a recorded HIV diagnosis in the HDSS-Clinic Link System (n = 685), described in further detail below, between August 1st, 2015, and December 31st, 2018. Individuals who were not diagnosed in one of the nine publicly funded health care facilities used by residents of the Agincourt HDSS (n = 251), had a viral load measurement below 400 copies/mL within seven days of diagnosis (n = 29) or migrated into the Agincourt HDSS after their first HIV diagnosis (n = 16) were excluded from the analysis to ensure participants were diagnosed and entered HIV care in an Agincourt HDSS facility.

### Data sources

We used data from the Agincourt HDSS-Clinic Link System, previously described, to determine population clinical outcomes [22–25]. Briefly, the Clinic Link System is a population-based clinical care database that covers consenting/assenting patients seeking HIV-specific services or chronic care in all nine publicly funded health care facilities used by study area residents. Data capturers supported by the Agincourt HDSS research team and stationed at each of the facilities since 2014 consent/assent patients seeking care on a daily basis. After obtaining written informed consent/assent clinical visit data and patient demographic data from physical patient files are captured in the Clinic Link System and linked to corresponding records in the Agincourt HDSS census database described below. Data capturers continually update clinical records data as individuals return for services. This dataset is highly robust and considered to be representative of individuals who have engaged in clinical care within the Agincourt HDSS since 2014.

Because the HDSS-Clinic Link System viral load data were occasionally missing, viral load measurements were supplemented using data from the South African National Health Laboratory Service. The South African National Health Laboratory Service provides HIV diagnostic services to approximately 80% of South Africans and conducted more than 5 million viral load tests across 16 laboratories in 2018 [26, 27].

Mortality and migration data were obtained from the Agincourt HDSS census database and linked to the HDSS-Clinic Link System data. The Agincourt HDSS database has been updated annually since 2000 and provides information on resident status and vital events such as migrations, births, and deaths [15].

### Measures

#### ***Linkage to care***

Individuals were considered linked to care on the first of the following dates: results delivered for CD4 testing after HIV diagnosis, a follow-up visit with an indication of HIV treatment delivery, or a CD4 or viral load test after HIV diagnosis.

#### ***Loss to follow-up***

Participants with no documented clinic visits for any given 90-day period following diagnosis were considered lost to follow-up (LTFU) on the first date the definition was met (i.e., the 90th day following the most recent visit date). This definition is consistent with a lapse in medication coverage, or “defaulting”, defined in the South African national HIV adherence guidelines, as medications are typically dispensed 90 days at a time [28]. We also considered a 180-day clinic visit lapse definition of LTFU as has been previously recommended (see Supplemental Tables 1–3) [29].

#### ***Treatment status***

Participants were considered on ART the first date of any HIV treatment medication pickup. Individuals who had a suppressed viral load measurement prior to the first recorded ART pickup date (n = 3) were considered on ART the same date as the suppressed viral load measurement.

#### ***Viral suppression status***

Consistent with our team’s existing work, viral load measurements less than 400 copies/mL were considered virally suppressed [30, 31].

#### ***Possible virologic failure***

Viral load measurements of 1000 copies/mL and above subsequent to a suppressed viral load measurement were considered indicative of possible virologic failure [32, 33].

#### ***Suboptimal care outcome***

Because death (n = 1) and possible virologic failure (n = 3) were uncommon in the study population overall, we combined the competing events of LTFU, death, and possible virologic failure in a “suboptimal care outcome” measure.

### Statistical analysis

**Fig. 1:**
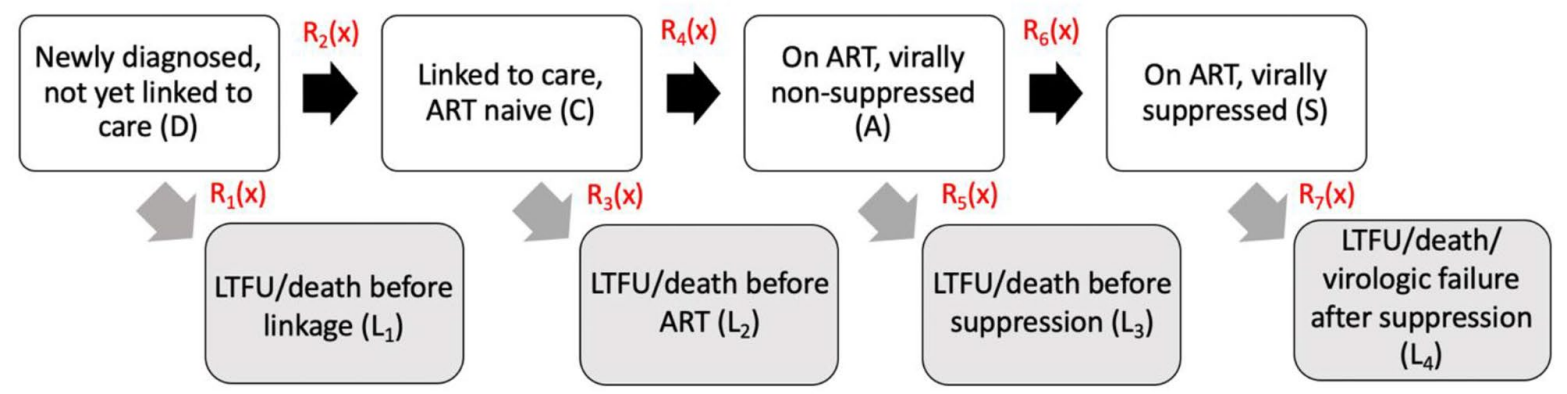
Conceptual framework for flow (arrows) through the longitudinal HIV care continuum stages (boxes) Abbreviations: ART-antiretroviral treatment initiation; LTFU- loss to follow-up

To characterize the longitudinal HIV care continuum in the study population we utilized analytic methods similar to those formalized by Lesko et al. [34]. These methods are similar to multistate analytic approaches and are designed to account for competing events and transitions into and out of multiple stages over time [3, 34]. First, we fit a Fine and Gray sub-distribution proportional hazards models with no covariates and used the Breslow estimator to calculate the cumulative incidence of seven distinct care continuum events specified in Table 1. Date of diagnosis served as the origin for each outcome of interest and administrative censoring occurred on day 365 following diagnosis or February 1, 2019. Competing events for each of the seven distinct outcomes are specified in Table 1. As detailed by Lesko et al., these outcomes were not of interest in and of themselves, but instead represent transitions between eight mutually exclusive stages of the longitudinal care continuum (Fig. 1) [35].

**Table 1:** Events of interest in the longitudinal HIV care continuum

Outcome	Definition	Competing event
Sub-optimal care outcome before linkage to care (R1)	Death date or absence of HIV clinic visit for any 90-day interval prior to care initiation	Linkage to care
Linkage to care (R_2_)	First visit when HIV-related medication or ART was dispensed, or viral load or CD4 count test was conducted	LTFU or death before linkage to care
Sub-optimal care outcome after linkage to care but before ART initiation (R_3_)	Death date or absence of HIV clinic visit for any 90-day interval between linkage to care and ART initiation	LTFU or death before linkage to care; ART initiation
ART initiation (R_4_)	First date of any HIV treatment medication pick up on or following date of HIV diagnosis	LTFU or death before linkage to care or ART initiation
Suboptimal care outcome after ART initiation but before viral suppression (R_5_)	Death date or absence of HIV clinic visit for any 90-day interval between ART initiation and achievement of viral suppression	LTFU or death before linkage to care or ART initiation; viral suppression
Viral suppression (R_6_)	Viral load < 400 copies/mL	LTFU or death before linkage care, ART initiation, or viral suppression
Sub-optimal care outcome after viral suppression (R_7_)	Death date or absence of HIV clinic visit for any 90-day interval after achievement of viral suppression or viral load $\ge$ 1000 copies/mL subsequent to viral suppression	LTFU or death before linkage to care, ART initiation, or viral suppression

Abbreviations: ART- antiretroviral therapy; LTFU- lost to follow-up

We estimated the proportion of the population in these eight mutually exclusive stages at each timepoint following diagnosis (i.e. on any day during the 365 days of follow-up). Events appearing in white in Fig. 1 (diagnosed with HIV but not yet linked to care; linked to care but ART naïve; on ART but virally non-suppressed; virally suppressed), represent the primary stages an individual progresses through in their care trajectory. Those appearing in gray (suboptimal care outcome before linkage to care; suboptimal care outcome after linkage but before ART initiation; suboptimal care outcome after ART initiation but before viral suppression; suboptimal care outcome after viral suppression) represent absorbing stages that preclude the individual from progressing through the remaining primary stages in the continuum.

The proportion of the population in each stage of the care continuum was estimated by adding and subtracting cumulative incidence curves as outlined in Table 2.

**Table 2:** Equations for estimating the proportion of individuals in each stage of the continuum at any given time point during study follow-up

**Primary stages**	
Diagnosed with HIV but not yet linked to care	P(D) = 1-R_1_(x)-R_2_(x)
Linked to care but ART naïve	P(C) = R_2_(x)-R_3_(x)-R_4_(x)
On ART but virally non-suppressed	P(A) = R_4_(x)- R_5_(x)-R_6_(x)
Virally suppressed	P(S) = R_6_(x)-R_7_(x)
**Absorbing stages**	
Suboptimal care outcome before linkage to care	P(L_1_) = R_1_(x)
Suboptimal care outcome after linkage but before ART initiation	P(L_2_) = R_3_(x)
Suboptimal care outcome after ART but before viral suppression	P(L_3_) = R_5_(x)
Suboptimal care outcome after viral suppression	P(L_4_) = R_7_(x)

Abbreviations: ART- antiretroviral treatment

These proportions are visually presented as a set of stacked curves that sum to one by design given the mutually exclusive nature of the eight care continuum stages. The area between adjacent curves represents the restricted (as each participant is followed for a maximum of one year and all outcomes may not be observed) mean time spent in that stage over the one-year follow-up period. Given individuals who experienced a sub-optimal care outcome (i.e., loss to follow-up, death, or possible virologic failure) were not permitted to reenter a primary stage following the occurrence of the sub-optimal outcome, estimates of the mean time spent linked to care but ART naïve; on ART but virally non-suppressed; and virally suppressed, represent the restricted mean time spent in each of these stages with no prior 90-or-more day gap in care.

Ultimately, we estimated (1) the proportion of participants in each of the eight stages of the continuum (four primary stages and four absorbing stages) and (2) the restricted mean time spent in each stage over the one-year period following diagnosis in the cohort overall and stratified by treatment era of diagnosis. We used inverse probability of treatment weights to account for meaningful differences in the distribution of sex and age at diagnosis between the two groups. Crude estimates are presented in Supplemental Tables 4 and 5. We calculated differences in outcomes among those diagnosed pre- (referent) and post-UTT implementation and estimated the 95% Wald confidence intervals (CI) using the standard error of estimates obtained from 300 non-parametric resamples of the data [36]. All analyses were conducted in SAS 9.4 (SAS Institute Inc., Cary, NC).

### Ethics

This study was approved by the University of North Carolina at Chapel Hill’s Institutional Review Board, the University of the Witwatersrand’s Human Research Ethics Committee, and the Mpumalanga Provincial Health Research Committee.

## Results

A total of 420 individuals were included. A majority were female (n = 389; 92.6%) and diagnosed after the adoption of UTT (n = 266; 63.3%) (Table 3). Median age at diagnosis was 22 (interquartile range [IQR]: 20–24) and median CD4 cell count at diagnosis or entry into care was 333 (IQR: 217–458) (Table 3).

**Table 3:** Baseline characteristics of study participants, stratified by treatment era of diagnosis

	Total (n = 420)	Pre-UTT (n = 154)	UTT (n = 266)
	n (%)	n (%)	n (%)
Sex			
Female	389 (92.6)	146 (94.8)	243 (91.4)
Male	31 (7.4)	8 (5.2)	23 (8.6)
Age at diagnosis (median/IQR)	22.2 (20.4–23.7)	22.3 (20.7–23.6)	22.2 (20.3–23.6)
CD4 cell count at diagnosis/entry into care (median/IQR)^†^	333 (217–458)	329 (200–438)	334 (221–483)
<200 cells/mm^3^	74 (22.5)	32 (24.8)	42 (21.0)
≥200 cells/mm^3^	255 (77.5)	97 (75.2)	158 (79.0)

Abbreviations: IQR- interquartile range; UTT- Universal Test and Treat

^†^missing baseline CD4 n = 91

At day 90 post-diagnosis, 81.8% of all participants had ever linked to care, 61.6% had ever initiated ART, and 5.9% had ever achieved viral suppression (S1 Figure); over one-third (33.8%) had become LTFU (S1 Figure). By 6 months post-diagnosis, 16.9% of all participants had ever achieved viral suppression (S1 Figure). By the end of the first year following diagnosis, 83.0% of all participants had ever linked to care, 69.4% had ever initiated ART, 30.9% had ever achieved viral suppression, 68.2% of all participants had become LTFU, 0.2% had died, and 0.7% had experienced virologic failure (S1 Figure). Participants spent a restricted mean time of 19.5 days (95% CI: 16.0, 23.0) between diagnosis and linkage to care; 29.4 days (95% CI: 25.6, 33.3) linked to care but ART-naive; 107.4 days (95% CI: 95.9, 118.8) on ART but virally non-suppressed; and 53.7 days (95% CI: 45.2, 62.2) virally suppressed (Table 4).

**Fig. 2:**
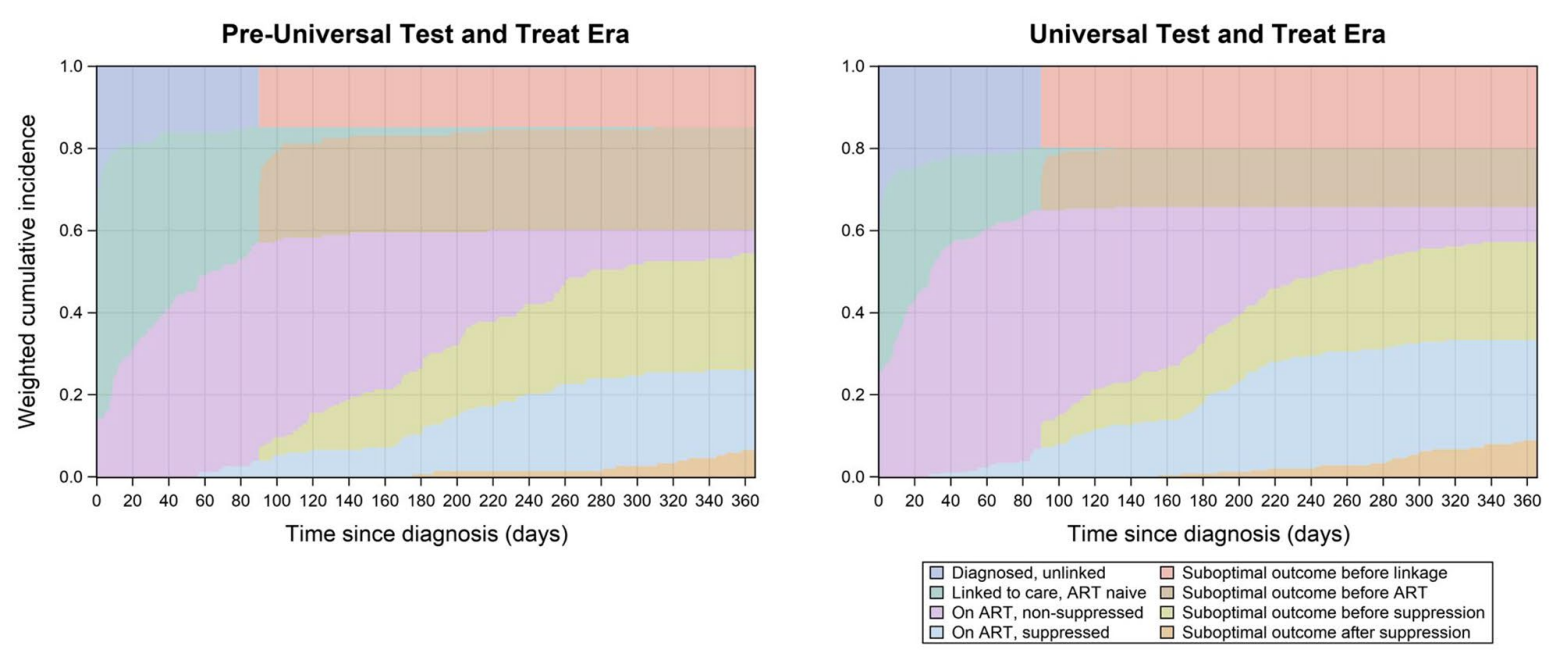
Cumulative incidence of HIV care outcomes over 1-year following diagnosis, stratified by treatment era of diagnosis Abbreviations: ART- antiretroviral treatment; LTFU- lost to follow-up; UTT- Universal Test and Treat Weighted to account for differences in age at diagnosis and sex by era of diagnosis

Approximately 25% of individuals diagnosed in the UTT era, compared to 15% of those diagnosed in the pre-UTT era initiated ART the same day they were diagnosed with HIV (Fig. 2). Individuals diagnosed in the UTT era initiated ART more quickly after linkage to care (restricted mean difference [MD] of time between linkage to care and ART initiation: -19.3; 95% CI: -27.7, -10.9), and spent more time over the one-year follow-up period on ART and virally suppressed when compared to those diagnosed in the pre-UTT era (MD of time spent on ART- virally non-suppressed: 12.7; 95% CI: -8.2, 33.7; MD of time spent virally suppressed: 17.7; 95% CI: 1.0, 34.4) (Table 5). By the end of follow-up, 85.2% of participants diagnosed in the pre-UTT era had ever linked to care, 60.1% had ever initiated ART, and 26.0% had ever achieved viral suppression (Fig. 2). Among those diagnosed in the UTT era, 80.2% had ever linked to care, 65.7% had ever initiated ART, and 33.3% had ever achieved viral suppression (Fig. 2). On the last day of follow-up, 5.7% (95% CI: 2.0, 9.4) of participants diagnosed in the pre-UTT era were on ART but non-suppressed, and 19.5% (95% CI: 13.1, 25.9) were virally suppressed (Table 6; Fig. 2). Among those diagnosed in the UTT era, 8.5% (95% CI: 4.7, 12.3) were on ART but non-suppressed, and 24.5% (95% CI: 19.1, 29.9) were virally suppressed (Table 6; Fig. 2). No meaningful differences were observed in the proportion of individuals in each of the primary stages of the care continuum at the end of the first year following diagnosis by treatment era of diagnosis (Table 6).

**Table 4:** Restricted mean time spent in each stage of care continuum over 1-year follow-up period and percent of individuals in each stage at end of follow-up

	Mean days (95% CI)	Percent (95% CI)
Primary stages		
Diagnosed with HIV but not yet linked to care	19.5 (16.0, 23.0)	0.1 (0.0, 0.3)
Linked to care but ART naïve	29.4 (25.6, 33.3)	0.0 (0.0, 0.0)
On ART but virally non-suppressed	107.4 (95.9, 118.8)	7.3 (4.5, 10.1)
Virally suppressed	53.7 (45.2, 62.2)	23.0 (18.7, 27.3)
**Absorbing stages**		
Suboptimal care outcome before linkage	49.7 (40.0, 59.4)	18.0 (14.4, 21.7)
Suboptimal care outcome after linkage but before ART	49.9 (39.9, 59.8)	18.5 (14.8, 22.3)
Suboptimal care outcome after ART but before suppression	48.8 (40.3, 57.2)	25.0 (20.8, 29.3)
Suboptimal care outcome after suppression	6.7 (4.1, 9.3)	7.9 (5.3, 10.5)

Abbreviations: ART- antiretroviral therapy; CI- confidence interval; UTT- Universal Test and Treat

**Table 5:** Differences in the restricted mean time spent in each stage of the HIV care continuum over 1-year follow-up period

	Pre-UTT wMean days^†^ (95% CI)	UTT wMean days^†^ (95% CI)	wDifference in mean days^†^ (95% CI)
**Primary stages**			
Diagnosed with HIV but not yet linked to care	16.9 (11.6, 22.1)	21.3 (16.7, 25.8)	4.4 (-2.5, 11.3)
Linked to care but ART naïve	41.5 (33.7, 49.3)	22.2 (18.6, 25.9)	-19.3 (-27.7, -10.9)
On ART but virally non-suppressed	99.4 (82.4, 116.4)	112.1 (98.0, 126.2)	12.7 (-8.2, 33.7)
Virally suppressed	42.2 (30.1, 54.4)	59.9 (48.2, 71.6)	17.7 (1.0, 34.4)
**Absorbing stages**			
Suboptimal care outcome before linkage	39.8 (26.0, 53.6)	54.0 (41.4, 66.7)	14.2 (-4.3, 32.7)
Suboptimal care outcome after linkage but before ART	66.1 (47.2, 85.1)	39.5 (28.8, 50.2)	-26.6 (-48.3, -5.0)
Suboptimal care outcome after ART but before suppression	54.0 (39.4, 68.6)	47.7 (36.7, 58.7)	-6.3 (-24.4, 11.8)
Suboptimal care outcome after suppression	5.0 (1.4, 8.7)	8.2 (4.5, 11.9)	3.2 (-2.0, 8.4)

Abbreviations: ART- antiretroviral therapy; CI- confidence interval; UTT- Universal Test and Treat; wMean- weighted mean

^†^Weighted to account for differences in age at diagnosis and sex by treatment era of diagnosis

Over 70% of participants diagnosed in the pre-UTT era, as compared to just over 60% in the post-UTT era had experienced a suboptimal care outcome by the end of the first year following diagnosis with HIV (Table 6; Fig. 2). Among those diagnosed in the pre-UTT era, sub-optimal outcomes were most common in the periods between linkage to care and ART initiation and ART initiation and viral suppression (Table 6). Among those diagnosed in the UTT era, sub-optimal outcomes were most common in the periods between diagnosis and linkage to care and ART initiation and viral suppression (Table 6).

**Table 6:** Proportion of participants in each stage of the HIV care continuum 1-year following diagnosis, stratified by treatment era of diagnosis

	Pre-UTT wPercent (95% CI)^†^	UTT wPercent (95% CI)^†^	wPercent difference (95% CI)^†^
**Primary stages**			
Diagnosed with HIV but not yet linked to care	0.4 (0.0, 0.7)	0.2 (0.0, 0.4)	-0.2 (-0.6, 0.3)
Linked to care but ART naïve	0.0 (-0.1, 0.1)	0.0 (0.0, 0.0)	0.0 (-0.1, 0.1)
On ART but virally non-suppressed	5.7 (2.0, 9.4)	8.5 (4.7, 12.3)	2.8 (-2.3, 7.9)
Virally suppressed	19.5 (13.1, 25.9)	24.5 (19.1, 29.9)	5.0 (-3.1, 13.1)
**Absorbing stages**			
Suboptimal care outcome before linkage	14.5 (9.3–19.6)	19.6 (14.9–24.3)	5.2 (-0.2, 12.0)
Suboptimal care outcome after linkage but before ART	25.0 (17.8–32.2)	14.4 (10.4–18.5)	-10.6 (-18.8, -2.4)
Suboptimal care outcome after ART but before suppression	28.4 (21.3–35.5)	23.9 (18.5–29.2)	-4.5 (-13.4, 4.4)
Suboptimal care outcome after suppression	6.5 (3.0-10.1)	8.9 (5.2–12.5)	2.3 (-2.7, 7.4)

Abbreviations: UTT- Universal Test and Treat; ART- antiretroviral therapy; CI- confidence interval; wPercent- weighted percent

^†^Weighted to account for differences in age at diagnosis and sex by treatment era of diagnosis

In analyses using the 180-day LTFU definition, differences in the restricted mean time spent on ART, but virally non-suppressed and virally suppressed and the proportion in each of these stages at the end of the one-year follow-up period were attenuated (S2-3 Tables). Differences in the restricted mean time spent diagnosed, but not yet linked to care and linked to care, but ART naïve were strengthened.

## Discussion

This study characterizes longitudinal HIV care continuum outcomes among YLHIV newly initiating HIV care in nine publicly funded health care facilities in rural South Africa pre- and post-implementation of the UTT policy. Overall, 83% of individuals had linked to care, 69% had initiated ART, and 31% had achieved viral suppression by one year following diagnosis. There were modest improvements in time spent on ART and virally suppressed among YLHIV diagnosed after the adoption of the UTT policy. However, the proportion of individuals diagnosed in the UTT era who had initiated ART, were retained in care, and were virally suppressed one year following diagnosis was well below the Joint United Nations’ Programme on HIV/AIDS’ 95-95-95 goal. Among those diagnosed in the UTT era, nearly one quarter experienced a sub-optimal care outcome in the period between ART initiation and viral suppression and around 20% experienced a sub-optimal outcome in the period between diagnosis and linkage to care.

Approximately 66% of individuals diagnosed during the UTT era initiated ART in the first year following diagnosis compared to just 60% of those diagnosed prior to UTT adoption. However, just 25% of those diagnosed during the UTT era initiated ART the same day as diagnosis as recommended under the UTT policy and the restricted mean time between linkage to care and ART initiation was over 22 days. The mean time spent virally suppressed among those diagnosed during the UTT era was 60 days, slightly higher than the approximately 40 days observed prior to UTT adoption. Nevertheless, just 25% of individuals diagnosed during the UTT era were suppressed at the end of follow-up representing a substantial gap in sustained retention on ART and viral suppression among YLHIV diagnosed in the era of UTT that must be urgently addressed. Differentiated, youth-friendly medical care and programs that target improvements in young people’s self-esteem, social support, and overall psychosocial well-being may be important to improving care outcomes such as viral suppression in this population [37–43]. A retrospective cohort study of youth initiating ART at 37 facilities in South Africa demonstrated just 19% of study participants receiving support services were non-suppressed 5-years after ART initiation compared to 37% of those who did not receive community based-services [43]. Care models such as these should be prioritized for South African YLHIV particularly in the critical first year following diagnosis.

Implementation of UTT appeared to influence the timing of sub-optimal care outcomes among YLHIV in this study. In both groups, sub-optimal outcomes were common in the period between ART initiation and achievement of viral suppression. However, those diagnosed in the pre-UTT era were more likely to experience a sub-optimal outcome in the period between linkage to care and ART initiation while those diagnosed in the UTT era were more likely to experience a sub-optimal outcome between diagnosis and linkage to care. Existing evidence suggests low ART readiness may impede retention in care following diagnosis in the UTT era [44]. For example, a study of adults immediately referred for ART in South Africa found that individuals who did not expect to receive a positive HIV diagnosis had significantly lower odds of ART readiness than others (adjusted odds ratio 0.26; 95% CI: 0.09, 0.78) and that the odds of linkage to care among those expressing treatment readiness were 2.97 times that in individuals who were not ready to initiate ART [44, 45]. Our data support the assertion that the period between diagnosis and linkage to care remains a critical time during which individuals may disengage from care, even after implementation of UTT. As such, improved counseling and linkage to care at the point of diagnosis with HIV are essential to the success of same-day ART initiation policies. Longitudinal counseling following diagnosis has been shown to improve linkage to care in other settings across sub-Saharan Africa [46, 47]. As just one example of the potential benefits of this type of intervention, in a cluster randomized controlled trial exploring the effects of a counseling intervention on treatment outcomes among people living with HIV in Uganda, those in the intervention arm were significantly more likely to link to care compared to those in the control arm [47].

Our findings have important implications for HIV policy and programming for South African YLHIV in the UTT era. First, increased resources in the UTT era should be spent on retention in care efforts for YLHIV, particularly in the periods between diagnosis and linkage to care and ART initiation and viral suppression as previously discussed. Second, increased attention must center on monitoring longitudinal care outcomes for this group, specifically. While the cross-sectional HIV care cascade [48] is useful in describing the proportion of individuals in each stage of the care continuum at a specific point in time, it provides a mere snapshot of the true patient experience [3, 49]. Longitudinal HIV care cascade data allows for a nuanced exploration of population-level outcomes over time [2, 3] and can aid in assessing the effectiveness of HIV care programs as individuals progress through each stage of the continuum. Ultimately, these cascades can be harnessed to identify specific gaps in treatment and care services at precise time points following diagnosis [2, 34].

This analysis had limitations. First, viral load monitoring is recommended once within the first six months following diagnosis with HIV, and just once annually after that under the South African National HIV Treatment guidelines [28]. Because we were interested in assessing HIV treatment outcomes in the first year following an individual’s diagnosis, most participants had one opportunity to achieve viral suppression during the study period. Among those who achieved viral suppression, true time to viral suppression may have been shorter but went uncaptured because of infrequent viral load monitoring. Similarly, virologic failure among individuals who achieved suppression may have been under-captured due to infrequent viral load monitoring. Misclassified person-time could subsequently result in biased effect estimates. Second, mortality and migration data were only accessible from 2014 to 2017. Individuals who died in 2018 and were not yet administratively censored or LTFU before the time of death would have misclassified person-time. Given just one participant died in the 2014–2017 follow-up period, this was a minor concern. Third, the Clinic Link System does not include data from individuals accessing private health care, or health care from clinics other than the nine publicly-funded facilities served by the study area. However, we do not expect this to represent a substantial issue as our prior work suggests a vast majority of study area residents seek care from the nine publicly funded facilities included in the HDSS Clinic Link System [19]. Lastly, the nature of the Clinic Link System prevented us from reliably ascertaining the reason for individuals’ LTFU. It is possible that individuals who were classified as LTFU actually transferred into care at a clinic outside the nine included health care facilities, as has been documented in other studies [24, 25, 50, 51]. Individuals who transferred care and were classified as LTFU may have progressed through additional stages in the HIV care continuum during the one-year follow-up period, though we do not expect differential migration with respect to treatment era.

## Conclusions

Implementation of the World Health Organization’s UTT policy yielded modest improvements in the time spent on ART and virally suppressed among South African YLHIV. However, with just 66% of YLHIV diagnosed in the UTT era initiating ART, and just 25% virally suppressed one year following diagnosis, meeting UNAIDS 95-95-95 targets remains a challenge. HIV treatment programs and policies for YLHIV in the UTT era should specifically center on improving longitudinal care outcome monitoring, including increased frequency of viral load monitoring, and retention in care in the periods immediately following diagnosis and ART initiation.

### Electronic supplementary material

Below is the link to the electronic supplementary material.

Supplementary Material 1

## Data Availability

The datasets analyzed during the current study are not publicly available due to the sensitive nature of HIV treatment and care data. A limited copy of the dataset may be made available from the corresponding author, Lindsey M. Filiatreau, on reasonable request.

## Abbreviations

ART: Anti-retroviral therapy
HDSS: Health and Socio-Demographic Surveillance System study area
HIV: Human-immunodeficiency virus
LTFU: Lost-to follow-up
UNAIDS: United Nations’ Joint Programme on HIV/AIDS
UTT: Universal test and treat policy
YLHIV: Youth living with HIV

## References

[1] Ehrenkranz P, Rosen S, Boulle A, Eaton JW, Ford N, Fox MP, et al. The revolving door of HIV care: revising the service delivery cascade to achieve the UNAIDS 95-95-95 goals. PLoS Med. 2021 May;18(5):e1003651. 10.1371/journal.pmed.1003651 PMC8186775 34029346

[2] Jose S, Delpech V, Howarth A, Burns F, Hill T, Porter K, et al. A continuum of HIV care describing mortality and loss to follow-up: a longitudinal cohort study. Lancet HIV. 2018 Jun;5(6):e301–8. 10.1016/S2352-3018(18)30048-1 PMC5990495 29893243

[3] Mody A, Glidden D v, Eshun-Wilson I, Sikombe K, Simbeza S, Mukamba N, et al. Longitudinal Care cascade outcomes among people eligible for antiretroviral therapy who are newly linking to care in Zambia: a multistate analysis. Clin Infect Dis. 2020 Dec;71(10):e561–70. 10.1093/cid/ciaa268 PMC7744998 32173743

[4] Beer L, Mattson CL, Bradley H, Shouse RL. Trends in ART prescription and viral suppression among HIV-positive young adults in care in the United States, 2009-2013. J Acquir Immune Defic Syndr (1988). 2017 Sep;76(1):e1–6. 10.1097/QAI.0000000000001427 PMC5552424 28489729

[5] Lee S, Kapogiannis BG, Allison S. Improving the youth HIV prevention and care continuums: the adolescent medicine trials network for HIV/AIDS interventions. JMIR Res Protoc. 2019 Mar;8(3):e12050. 10.2196/12050 PMC6454340 30912750

[6] Zanoni BC, Mayer KH. The adolescent and young adult HIV cascade of care in the United States: exaggerated health disparities. AIDS Patient Care STDS. 2014 Mar;28(3):128–35. 10.1089/apc.2013.0345 PMC3948479 24601734

[7] Enane LA, Davies MA, Leroy V, Edmonds A, Apondi E, Adedimeji A, et al. Traversing the cascade: urgent research priorities for implementing the “treat all” strategy for children and adolescents living with HIV in sub-Saharan Africa. J Virus Erad. 2018;4(Suppl 2):40–6. 10.1016/S2055-6640(20)30344-7 PMC6248846 30515313

[8] UNAIDS. Ending the AIDS epidemic for adolescents, with adolescents. Geneva, Switzerland; 2016.

[9] Slogrove AL, Sohn AH. The global epidemiology of adolescents living with HIV: time for more granular data to improve adolescent health outcomes. Curr Opin HIV AIDS. 2018;13:170–8. 10.1097/COH.0000000000000449 PMC5929160 29432227

[10] Zanoni BC, Archary M, Buchan S, Katz IT, Haberer JE. Systematic review and meta-analysis of the adolescent HIV continuum of care in South Africa: the Cresting Wave. BMJ Glob Health. 2016;1(e000004). 10.1136/bmjgh-2015-000004 PMC5321340 28588949

[11] Lippman SA, el Ayadi AM, Grignon JS, Puren A, Liegler T, Venter WDF, et al. Improvements in the South African HIV care cascade: findings on 90-90-90 targets from successive population-representative surveys in North West Province. J Int AIDS Soc. 2019 Jun;22(6). 10.1002/jia2.25295 PMC6562149 31190460

[12] Onoya D, Sineke T, Hendrickson C, Mokhele I, Maskew M, Long LC, et al. Impact of the test and treat policy on delays in antiretroviral therapy initiation among adult HIV positive patients from six clinics in Johannesburg, South Africa: Results from a prospective cohort study. BMJ Open. 2020;10:e030228. 10.1136/bmjopen-2019-030228 PMC7170559 32213514

[13] Pettifor A, Filiatreau L, Delany-Moretlwe S. Time to strengthen HIV treatment and prevention for youth. Lancet HIV. 2019 Oct;6(11). 10.1016/S2352-3018(19)30232-2 31585837

[14] Davies MA, Pinto J. Targeting 90-90-90 - Don’t leave children and adolescents behind. J Int AIDS Soc. 2015;18(Suppl 6):20745. 10.7448/IAS.18.7.20745 PMC4670834 26639121

[15] Kahn K, Collinson MA, Xavier G?mez-oliv? F, Mokoena O, Twine R, Mee P, et al. Profile: agincourt health and socio-demographic surveillance system. Int J Epidemiol. 2012 Aug;41(4):988–1001. 10.1093/ije/dys115 PMC3429877 22933647

[16] MRC/Wits Agincourt Research Unit. Research findings ? MRC/Wits agincourt unit. 2014. Available from: https://www.agincourt.co.za/?page_id=1911

[17] Gómez-Olivé FX, Angotti N, Houle B, Klipstein-Grobusch K, Kabudula C, Menken J, et al. Prevalence of HIV among those 15 and older in rural South Africa. AIDS Care. 2013;25(9):1122–8. 10.1080/09540121.2012.750710 PMC3778517 23311396

[18] Collinson MA, White MJ, Bocquier P, McGarvey ST, Afolabi SA, Clark SJ, et al. Migration and the epidemiological transition: insights from the agincourt sub-district of northeast South Africa. Glob Health Action. 2014;7(SUPP.1). 10.3402/gha.v7.23514 PMC4028907 24848656

[19] Ginsburg C, Collinson MA, Gómez-Olivé FX, Gross M, Harawa S, Lurie MN, et al. Internal migration and health in South Africa: determinants of healthcare utilisation in a young adult cohort. BMC Public Health. 2021 Dec;21(1):1–15. 10.1186/s12889-021-10590-6 PMC7981972 33743663

[20] National Department of Health Republic of South Africa. National consolidated guidelines for the prevention of mother-to-child transmission of HIV (PMTCT) and the management of HIV in children, adolescents and adults. Pretoria, South Africa; 2015.

[21] West R, Leslie H, Gómez-Olivé FX, Kahn K, Twine R, Filiatreau L, et al. Using multiple modes of assessment to measure patient experience in public clinics in rural Mpumalanga, South Afria. In: South African AIDS Conference. Durban, South Africa; 2019.

[22] Kabudula CW, Clark BD, Gómez-Olivé FX, Tollman S, Menken J, Reniers G. The promise of record linkage for assessing the uptake of health services in resource constrained settings: a pilot study from South Africa. BMC Med Res Methodol. 2014 Dec;14(1):71. 10.1186/1471-2288-14-71 PMC4041350 24884457

[23] Lippman SA, Pettifor A, Rebombo D, Julien A, Wagner RG, Kang Dufour MS, et al. Evaluation of the Tsima community mobilization intervention to improve engagement in HIV testing and care in South Africa: study protocol for a cluster randomized trial. Implement Sci. 2017 Jan;12(1):1–13. 10.1186/s13012-016-0541-0 PMC5240325 28095904

[24] Etoori D, Gomez-Olive FX, Reniers G, Rice B, Renju J, Kabudula CW, et al. Outcomes after being lost to follow-up differ for pregnant and postpartum women when compared with the general HIV treatment population in rural South Africa. J Acquir Immune Defic Syndr. 2020 Oct;85(2):127–37. 10.1097/QAI.0000000000002413 PMC7495979 32520907

[25] Etoori D, Wringe A, Kabudula CW, Renju J, Rice B, Gomez-Olive FX, et al. Misreporting of patient outcomes in the South African National HIV treatment database: consequences for programme planning, monitoring, and evaluation. Front Public Health. 2020 Apr;8:100. 10.3389/fpubh.2020.00100 PMC7154050 32318534

[26] Sherman GG, Mazanderani AH, Barron P, Bhardwaj S, Niit R, Okobi M, et al. Toward elimination of mother-to-child transmission of HIV in South Africa: how best to monitor early infant infections within the Prevention of Mother-to-Child Transmission Program. J Glob Health. 2017 Apr;7(1). 10.7189/jogh.07.010701 PMC5441442 28567281

[27] Girdwood SJ, Crompton T, Sharma M, Dorward J, Garrett N, Drain PK, et al. Cost-effectiveness of adoption strategies for point of care HIV viral load monitoring in South Africa. EClinicalMedicine. 2020 Nov;28:100607. 10.1016/j.eclinm.2020.100607 PMC7700965 33294817

[28] Department of Health Republic of South Africa. Adherence guidelines for HIV, TB and NCDs. Policy and service guidelines for linkage to care, adherence to treatment and retention in care. Pretoria, South Africa; 2016. Available from: https://www.nacosa.org.za/wp-content/uploads/2016/11/Integrated-Adherence-Guidelines-NDOH.pdf

[29] Phillips TK, Orrell C, Brittain K, Zerbe A, Abrams EJ, Myer L. Measuring retention in HIV care: the impact of data sources and definitions using routine data in South Africa. AIDS. 2020 Apr;34(5):749. 10.1097/QAD.0000000000002478 PMC7109335 32004202

[30] Moosa A, Gengiah TN, Lewis L, Naidoo K. Long-term adherence to antiretroviral therapy in a South African adult patient cohort: a retrospective study. BMC Infect Dis. 2019 Sep;19(1):775. 10.1186/s12879-019-4410-8 PMC6727323 31488063

[31] Kharsany ABM, Cawood C, Lewis L, Yende-Zuma N, Khanyile D, Puren A, et al. Trends in HIV prevention, treatment, and incidence in a hyperendemic area of KwaZulu-Natal, South Africa. JAMA Netw Open. 2019 Nov;2(11):e1914378. 10.1001/jamanetworkopen.2019.14378 PMC6826647 31675082

[32] Kiweewa F, Esber A, Musingye E, Reed D, Crowell TA, Cham F, et al. HIV virologic failure and its predictors among HIV-infected adults on antiretroviral therapy in the African Cohort Study. PLoS One. 2019 Feb;14(2):e0211344. 10.1371/journal.pone.0211344 PMC6363169 30721233

[33] Laprise C, De Pokomandy A, Baril JG, Dufresne S, Trottier H. Virologic failure following persistent low-level viremia in a cohort of HIV-positive patients: results from 12 years of observation. Clin Infect Dis. 2013 Nov;57(10):1489–96. 10.1093/cid/cit529 23946221

[34] Lesko CR, Edwards JK, Moore RD, Lau B. A longitudinal, HIV care continuum: 10-year restricted mean time in each care continuum stage after enrollment in care, by history of IDU. AIDS. 2016;30(14):2227–34. 10.1097/QAD.0000000000001183 PMC5063502 27314178

[35] Lesko CR, Edwards JK, Hanna DB, Mayor AM, Silverberg MJ, Horberg M, et al. Longitudinal HIV care outcomes by gender identity in the United States. AIDS. 2022 Nov;36(13):1841–9. 10.1097/QAD.0000000000003339 PMC9529804 35876653

[36] Efron B, Tibshirani R. An Introduction to the Bootstrap. 1st ed. Chapman and Hall/CRC; 1994.

[37] Willis N, Napei T, Armstrong A, Jackson H, Apollo T, Mushavi A, et al. Zvandiri-Bringing a differentiated service delivery program to scale for children, adolescents, and young people in Zimbabwe. J Acquir Immune Defic Syndr. 2018;78 Suppl 2:S115–23. 10.1097/QAI.0000000000001737 29994833

[38] Reif LK, McNairy ML, Lamb MR, Fayorsey R, Elul B. Youth-friendly services and differentiated models of care are needed to improve outcomes for young people living with HIV. Curr Opin HIV AIDS. 2018 May 1;13(3):249–56. 10.1097/COH.0000000000000454 29432230

[39] Ruria EC, Masaba R, Kose J, Woelk G, Mwangi E, Matu L, et al. Optimizing linkage to care and initiation and retention on treatment of adolescents with newly diagnosed HIV infection. AIDS. 2017 Jul;31:S253–60. 10.1097/QAD.0000000000001538 PMC5497791 28665883

[40] Lamb MR, Fayorsey R, Nuwagaba-Biribonwoha H, Viola V, Mutabazi V, Alwar T, et al. High attrition before and after ART initiation among youth (15?24 years of age) enrolled in HIV care. AIDS. 2014 Feb;28(4):559–68. 10.1097/QAD.0000000000000054 PMC4517438 24076661

[41] Bhana A, Mellins CA, Petersen I, Alicea S, Myeza N, Holst H, et al. The VUKA family program: piloting a family-based psychosocial intervention to promote health and mental health among HIV infected early adolescents in South Africa. AIDS Care. 2014 Jan;26(1):1–11. 10.1080/09540121.2013.806770 PMC3838445 23767772

[42] Casale M, Carlqvist A, Cluver L. Recent interventions to improve retention in hiv care and adherence to antiretroviral treatment among adolescents and youth: a systematic review. AIDS Patient Care STDS. 2019 Jun;33(6):237. 10.1089/apc.2018.0320 PMC6588099 31166783

[43] Fatti G, Jackson D, Goga AE, Shaikh N, Eley B, Nachega JB, et al. The effectiveness and cost-effectiveness of community-based support for adolescents receiving antiretroviral treatment: an operational research study in South Africa. J Int AIDS Soc. 2018 Feb 1;21(Suppl 1). 10.1002/jia2.25041 PMC5978711 29485714

[44] Maughan-Brown B, Harrison A, Gal?rraga O, Kuo C, Smith P, Bekker LG, et al. Factors affecting linkage to HIV care and ART initiation following referral for ART by a mobile health clinic in South Africa: evidence from a multimethod study. J Behav Med. 2019 Oct;42(5):883–97. 10.1007/s10865-018-0005-x PMC6625943 30635862

[45] Maughan-Brown B, Smith P, Kuo C, Harrison A, Lurie MN, Bekker LG, et al. Readiness for antiretroviral therapy: implications for linking HIV-infected individuals to care and treatment. AIDS Behav. 2018 Mar;22(3):691–700. 10.1007/s10461-017-1834-2 PMC5785568 28752353

[46] Herce ME, Chi BH, Liao RC, Hoffmann CJ. Re-thinking linkage to care in the era of universal test and treat: insights from implementation and behavioral science for achieving the second 90. AIDS Behav. 2019 Sep;23(2):120–8. 10.1007/s10461-019-02541-5 PMC6773672 31161462

[47] Ruzagira E, Grosskurth H, Kamali A, Baisley K. Brief counselling after home-based HIV counselling and testing strongly increases linkage to care: a cluster-randomized trial in Uganda. J Int AIDS Soc. 2017 Oct 1;20(2):e25014. 10.1002/jia2.25014 PMC5810339 29052344

[48] Gardner EM, McLees MP, Steiner JF, del Rio C, Burman WJ. The spectrum of engagement in HIV care and its relevance to test-and-treat strategies for prevention of HIV infection. Clin Infect Dis. 2011 Mar;52(6):793–800. 10.1093/cid/ciq243 PMC3106261 21367734

[49] Nash D, Robertson MK. How to evolve the response to the global HIV epidemic with new metrics and targets based on pre-treatment CD4 counts. Curr Opin HIV AIDS. 2019;16:304–13. 10.1007/s11904-019-00452-7 PMC10938289 31278620

[50] Geng EH, Odeny TA, Lyamuya R, Nakiwogga-Muwanga A, Diero L, Bwana M, et al. Retention in care and patient-reported reasons for undocumented transfer or stopping care among HIV-infected patients on antiretroviral therapy in Eastern Africa: application of a sampling-based approach. Clin Infect Dis. 2016;62(7):935–44. 10.1093/cid/civ1004 PMC4787603 26679625

[51] Padian NS, Holmes CB. Where have all the patients gone? Lancet HIV. 2015 Mar;2(3):e78–9. 10.1016/S2352-3018(15)00015-6 26424547

