# Supplementary material for: Understanding the effects of universal test and treat on longitudinal HIV care outcomes among South African youth: a retrospective cohort study
Source: BMC Public Health. 2023 Sep 5;23:1724. doi: 10.1186/s12889-023-16353-9 (PMC10478421; doi:10.1186/s12889-023-16353-9)
Supplement: Supplementary file 1 — Supplementary Material 1 [file 12889_2023_16353_MOESM1_ESM.docx]

**Supplemental Digital Content**

**Supplemental Table 1. Time spent in each stage of care continuum over 1-year follow-up period and percent of individuals in each stage at end of follow-up^a^**

|  | **Mean days**  **(95% CI)** | **Percent (95% CI)** |
| --- | --- | --- |
| Diagnosed with HIV, not yet linked | 35.3 (28.9, 41.8) | 0.1 (0.0, 0.3) |
| Suboptimal treatment outcome before linkage | 31.2 (25.3, 37.1) | 16.9 (13.5, 20.2) |
| Linked to care, ART naïve | 44.2 (37.6, 50.9) | 0.2 (-0.2, 0.7) |
| Suboptimal treatment outcome before ART | 23.2 (17.4, 28.9) | 13.3 (9.9, 16.7) |
| On ART, non-suppressed | 142.0 (130.3, 153.8) | 15.6 (11.9, 19.3) |
| Suboptimal treatment outcome before suppression | 18.9 (14.4, 23.3) | 15.9 (12.2, 19.6) |
| On ART, suppressed | 67.9 (58.4, 77.4) | 35.4 (30.5, 40.4) |
| Suboptimal treatment outcome after suppression | 2.3 (1.0, 3.7) | 2.5 (1.0, 4.0) |

ART- antiretroviral therapy; CI- confidence interval; UTT- Universal Test and Treat

^a^Loss to follow up defined as no documented clinic visits for any given 180-day period following diagnosis

**Supplemental Table 2. Time spent in each stage of the HIV care continuum over 1-year follow-up period by treatment era of diagnosis^a^**

|  | **Pre-UTT**  **Mean days**  **(95% CI)** | **UTT**  **Mean days**  **(95% CI)** | **Difference in mean days**  **(95% CI)** |
| --- | --- | --- | --- |
| **Crude** |  |  |  |
| Diagnosed with HIV, not yet linked | 30.7 (20.3, 41.1) | 38.6 (30.3, 47.0) | 8.0 (-5.6, 21.5) |
| Suboptimal treatment outcome before linkage | 27.4 (17.9, 37.0) | 33.3 (25.6, 41.0) | 5.8 (-6.8, 18.4) |
| Linked to care, ART naïve | 57.7 (44.9, 70.4) | 36.1 (29.0, 43.2) | -21.6, -35.9, -7.2) |
| Suboptimal treatment outcome before ART | 24.6 (15.3, 34.0) | 22.4 (15.4, 29.4) | -2.2 (-13.6, 9.2) |
| On ART, non-suppressed | 138.6 (119.6, 157.6) | 143.5 (128.9, 158.1) | 4.9 (-18.6, 28.4) |
| Suboptimal treatment outcome before suppression | 21.4 (14.5, 28.3) | 17.6 (12.3, 22.8) | -3.8 (-12.2, 4.5) |
| On ART, suppressed | 62.8 (48.5, 77.2) | 70.8 (59.1, 82.5) | 8.0 (-9.8, 25.7) |
| Suboptimal treatment outcome after suppression | 1.7 (0.2, 3.3) | 2.7 (0.7, 4.7) | 1.0 (-1.7, 3.6) |
| **Weighted^b^** |  |  |  |
| Diagnosed with HIV, not yet linked | 29.9 (19.7, 40.0) | 38.3 (30.0, 46.6) | 8.4 (-4.9, 21.7) |
| Suboptimal treatment outcome before linkage | 26.8 (17.5, 36.0) | 32.9 (25.2, 40.5) | 6.1 (-6.2, 18.5) |
| Linked to care, ART naïve | 57.9 (45.0, 70.9) | 35.8 (28.7, 42.9) | -22.2 (-36.7, -7.6) |
| Suboptimal treatment outcome before ART | 24.7 (15.3, 34.0) | 22.1 (15.2, 29.1) | -2.5 (-13.9, 8.8) |
| On ART, non-suppressed | 139.7 (120.7, 158.8) | 144.2 (129.6, 158.9) | 4.5 (-19.2, 28.2) |
| Suboptimal treatment outcome before suppression | 22.2 (15.1, 29.3) | 17.8 (12.4, 23.1) | -4.4 (-13.1, 4.2) |
| On ART, suppressed | 62.0 (47.6, 76.3) | 71.1 (59.3, 82.9) | 9.2 (-8.5, 26.9) |
| Suboptimal treatment outcome after suppression | 1.8 (0.2, 3.5) | 2.8 (0.7, 5.0) | 1.0 (-1.8, 3.8) |

Abbreviations: UTT- Universal Test and Treat; ART- antiretroviral therapy; CI- confidence interval

^a^Loss to follow up defined as no documented clinic visits for any given 180-day period following diagnosis

^b^Weighted to account for differences in age at diagnosis and sex by treatment era of diagnosis

**Supplemental Table 3. Proportion of participants in each stage of the HIV care continuum 1-year following diagnosis by treatment era of diagnosis^a^**

|  | **Pre-UTT**  Percent (95% CI) | **UTT**  Percent (95% CI) | **Percent difference**  (95% CI) |
| --- | --- | --- | --- |
| **Crude** |  |  |  |
| Diagnosed with HIV, not yet linked | 0.4 (0.0, 0.8) | 0.2 (0.0, 0.5) | -0.2 (-0.6, 0.3) |
| Suboptimal treatment outcome before linkage | 14.8 (9.3, 20.4) | 18.0 (13.7, 22.2) | 3.1 (-3.9, 10.2) |
| Linked to care, not on ART | 0.6 (-0.6, 1.9) | 0.0 (0.0, 0.0) | -0.6 (-1.9, 0.6) |
| Suboptimal treatment outcome before ART | 14.2 (8.4, 20.0) | 12.8 (8.6, 16.9) | -1.5 (-8.5, 5.5) |
| On ART, non-suppressed | 14.2 (8.8, 19.6) | 16.6 (11.9, 21.2) | 2.3 (-4.4, 9.0) |
| Suboptimal treatment outcome before suppression | 19.4 (13.1, 25.7) | 13.5 (9.3, 17.7) | -5.9 (-13.4, 1.5) |
| On ART, suppressed | 33.7 (25.9, 41.4) | 36.6 (30.5, 42.6) | 2.6 (0.1, 5.1) |
| Suboptimal treatment outcome after suppression | 2.6 (0.0, 5.1) | 2.4 (0.6, 4.3) | -0.1 (-3.3, 3.1) |
| **Weighted** |  |  |  |
| Diagnosed with HIV, not yet in care | 0.3 (0.0, 0.7) | 0.2 (0.0, 0.5) | -0.1 (-0.5, 0.3) |
| Suboptimal treatment outcome before linkage | 14.5 (9.1, 19.9) | 17.8 (13.5, 22.0) | 3.3 (-3.6, 10.2) |
| Linked to care, not on ART | 0.6 (-0.6, 1.9) | 0.0 (0.0, 0.0) | -0.6 (-1.9, 0.6) |
| Suboptimal treatment outcome before ART | 14.4 (8.5, 20.2) | 12.6 (8.5, 16.6) | -1.8 (-8.9, 5.2) |
| On ART, non-suppressed | 14.1 (8.7, 19.5) | 16.7 (12.0, 21.3) | 2.6 (-4.1, 9.2) |
| Suboptimal treatment outcome before suppression | 20.3 (13.7, 26.9) | 13.7 (9.4, 17.9) | -6.6 (-14.3, 1.1) |
| On ART, suppressed | 33.1 (25.3, 40.9) | 36.5 (30.4, 42.6) | 3.4 (-6.2, 13.1) |
| Suboptimal treatment outcome after suppression | 2.7 (0.0, 5.3) | 2.5 (0.6, 4.5) | -0.1, (-3.4, 3.2) |

ART- antiretroviral therapy; CI- confidence interval; UTT- Universal Test and Treat

^a^Loss to follow up defined as no documented clinic visits for any given 180-day period following diagnosis

^b^Weighted to account for differences in age at diagnosis and sex by treatment era of diagnosis

**Supplemental Table 4. Crude time spent in each stage of the HIV care continuum over 1-year follow-up period by treatment era of diagnosis^a^**

|  | **Pre-UTT**  **Mean days**  **(95% CI)** | **UTT**  **Mean days**  **(95% CI)** | **Difference in mean days**  **(95% CI)** |
| --- | --- | --- | --- |
| Diagnosed with HIV (not yet linked) | 17.3 (11.9, 22.7) | 21.4 (16.8, 26.0) | 4.1 (-2.9, 11.2) |
| Suboptimal treatment outcome before linkage | 40.8 (26.7, 55.0) | 54.7 (42.0, 67.3) | 13.8 (-5.0, 32.7) |
| Linked to care (ART naïve) | 41.3 (33.6, 49.0) | 22.3 (18.7, 26.0) | -18.9 (-27.3, -10.6) |
| Suboptimal treatment outcome before ART | 66.9 (47.9, 85.9) | 40.0 (29.2, 50.8) | -26.9 (-48.7, -5.1) |
| On ART (non-suppressed) | 99.2 (82.2, 116.3) | 111.8 (97.6, 125.9) | 12.5 (-8.5, 33.5) |
| Suboptimal treatment outcome before suppression | 51.3 (37.4, 65.2) | 47.2 (36.3, 58.1) | -4.1 (-21.5, 13.3) |
| Virally suppressed | 43.2 (31.0, 55.5) | 59.6 (47.9, 71.3) | 16.4 (-0.4, 33.1) |
| Suboptimal treatment outcome after suppression | 4.9 (1.4, 8.4) | 8.0 (4.4, 11.5) | 3.1 (-1.9, 8.0) |

Abbreviations: ART- antiretroviral therapy; CI- confidence interval; UTT- Universal Test and Treat

^a^Loss to follow up defined as no documented clinic visits for any given 90-day period following diagnosis

**Supplemental Table 5. Crude proportion of participants in each stage of the HIV care continuum 1-year following diagnosis by treatment era of diagnosis^a^**

|  | **Pre-UTT**  Percent (95% CI) | **UTT**  Percent (95% CI) | **Percent difference**  (95% CI) |
| --- | --- | --- | --- |
| **Crude** |  |  |  |
| Diagnosed with HIV (not yet linked) | 0.4 (0.0, 0.7) | 0.2 (0.0, 0.4) | -0.2 (-0.6, 0.3) |
| Suboptimal treatment outcome before linkage | 14.8 (9.6, 20.1) | 19.9 (15.2, 24.6) | 5.0 (-2.0, 12.0) |
| Linked to care (ART naïve) | 0.0 (-0.1, 0.1) | 0.0 (0.0, 0.0) | 0.0 (-0.1, 0.1) |
| Suboptimal treatment outcome before ART | 25.2 (18.0-32.4) | 14.6 (10.6-18.7) | -10.6 (-18.8, -2.4) |
| On ART (non-suppressed) | 5.8 (2.1, 9.6) | 8.5 (4.7, 12.3) | 2.6 (-2.5, 7.8) |
| Suboptimal treatment outcome before suppression | 27.2 (20.3-34.1) | 23.6 (18.2-28.9) | -3.6 (-12.3, 5.0) |
| Virally suppressed | 20.1 (13.6, 26.5) | 24.5 (19.1, 29.9) | 4.5 (-3.7, 12.7) |
| Suboptimal treatment outcome after suppression | 6.5 (3.0-10.0) | 8.7 (5.2-12.3) | 2.2 (-2.7, 7.2) |

Abbreviations: UTT- Universal Test and Treat; ART- antiretroviral therapy; CI- confidence interval

^a^Loss to follow up defined as no documented clinic visits for any given 90-day period following diagnosis

**
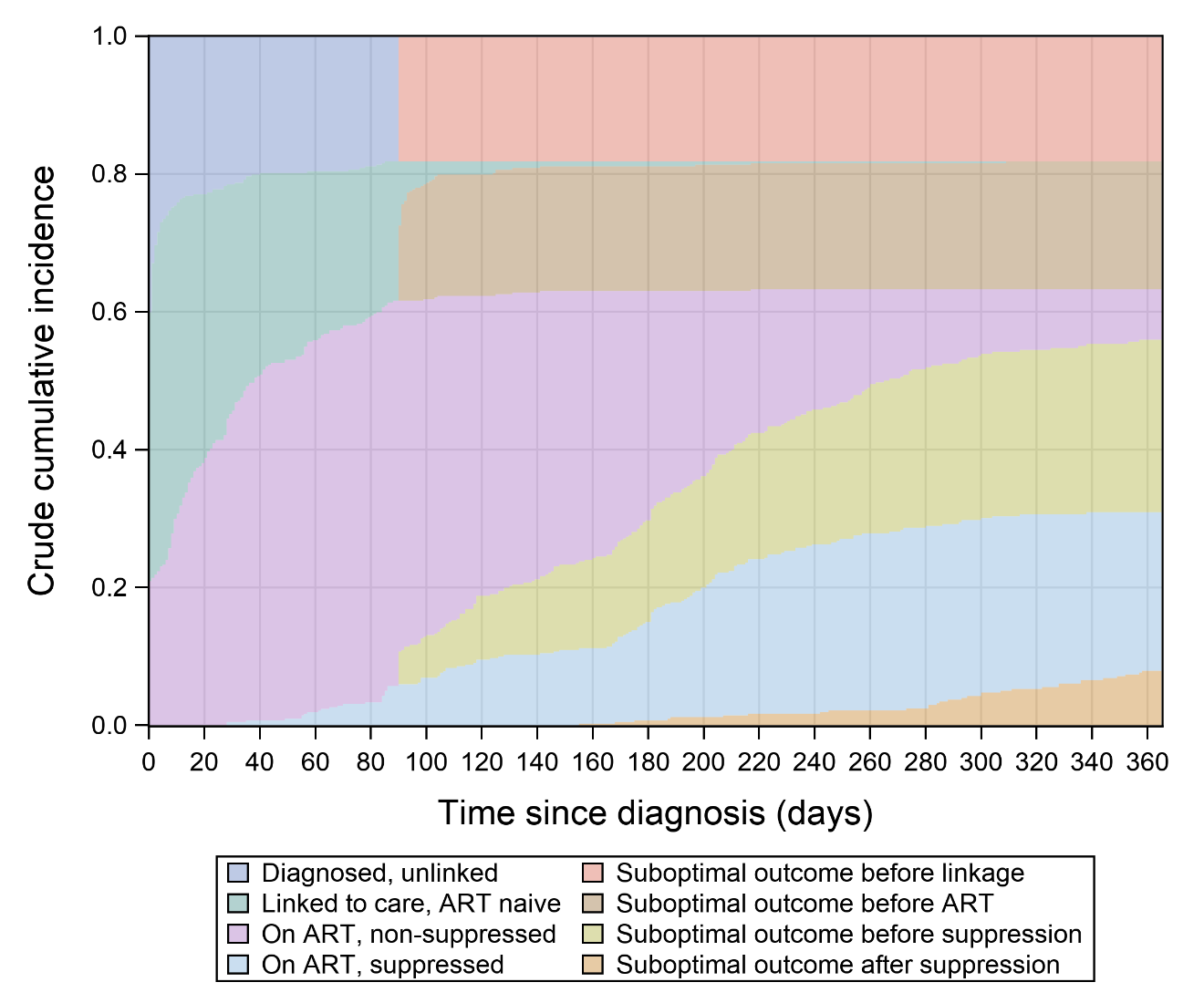
**

**Supplemental Figure 1. Crude cumulative incidence of HIV care outcomes over the 1-year follow-up period in study population overall with loss to follow up defined as no documented clinic visits for any given 90-day period following diagnosis**

ART- antiretroviral treatment; LTFU- lost to follow-up; UTT- Universal Test and Treat
